# The Use of Non-Invasive Brain Stimulation Techniques in Subjects with Parkinson’s Disease and Mild Cognitive Impairment: A Systematic Review

**DOI:** 10.3390/brainsci16030325

**Published:** 2026-03-19

**Authors:** Davide Mazzara, Angelo Torrente, Paolo Alonge, Giulia Gerardi, Anna Renda, Roberto Monastero

**Affiliations:** 1 Department of Biomedicine, Neuroscience and Advanced Diagnostics (Bi.N.D.), University of Palermo, 90127 Palermo, Italy; davide.mazzara29@gmail.com (D.M.); angelo.torrente@unipa.it (A.T.); paolo.alonge01@unipa.it (P.A.); giulia.gerardi@community.unipa.it (G.G.); anna.renda1982@gmail.com (A.R.); 2 IRCCS Centro Neurolesi Bonino Pulejo, 98124 Messina, Italy

**Keywords:** Parkinson’s disease, PD, mild cognitive impairment, MCI, non-invasive brain stimulation, transcranial magnetic stimulation, TMS, transcranial electrical stimulation, tDCS, cognitive improvement

## Abstract

**Highlights:**

**What are the main findings?**

• Non-invasive brain stimulation (NIBS), particularly intermittent theta burst stimulation and transcranial direct current stimulation targeting the dorsolateral prefrontal cortex, shows some efficacy in improving episodic memory and global cognition in patients with Parkinson’s disease (PD) and mild cognitive impairment (MCI).
• The detection of cognitive improvement is highly dependent on the sensitivity of assessment tools, with the Montreal Cognitive Assessment and the Repeatable Battery for the Assessment of Neuropsychological Status having superior results compared to tests of global cognition such as the Mini-Mental State Examination.

**What are the implications of the main findings?**

• NIBS represents a promising non-pharmacological add-on therapy for specific cognitive deficits in PD.
• Future clinical trials should prioritize combined interventions (stimulation plus cognitive training) and include medium-term follow-up to capture delayed neuroplastic effects on executive functions.

**Abstract:**

**Background/Objectives:** Mild cognitive impairment (MCI) is common in Parkinson’s disease (PD) and significantly impacts quality of life. Non-invasive brain stimulation (NIBS) techniques have emerged as potential therapeutic interventions. This systematic review analyzes the current evidence regarding the efficacy of Transcranial magnetic stimulation (TMS) and transcranial electrical stimulation (tES) on cognitive domains in patients with PD-MCI. **Methods:** A systematic search was conducted across the PubMed, Scopus, Web of Science, and Medline Ultimate databases up to 20 November 2025. We included studies investigating the effects of NIBS compared to sham stimulation on neuropsychological outcomes in diagnosed PD-MCI patients. **Results:** Eight studies involving different stimulation protocols were included. Interventions primarily used TMS or tES targeting the left dorsolateral prefrontal cortex (DLPFC). Episodic memory and global cognition were the most responsive domains, assessed with specific neuropsychological scales. Findings for executive functions and attention were heterogeneous, while visuospatial abilities generally showed limited immediate response. **Conclusions:** NIBS represents a promising but low-certainty-evidence adjunctive therapy for PD-MCI, with improvements found in memory and global cognition. Future research should prioritize larger sample sizes, combined interventions (NIBS plus cognitive rehabilitation), and extended follow-ups to evaluate long-term neuroplasticity.

## 1. Introduction

Parkinson’s Disease (PD) is a neurodegenerative disorder characterized by motor and non-motor symptoms. Age is the most important risk factor for developing PD, as the prevalence increases with increasing age, being around 1% among individuals aged 60 and over, and up to 3–5% among those aged 85 and over [1]. The disease is more common in men than in women, with a pooled male-to-female ratio of 1.6 [2]. PD shows an incidence of approximately 15–20 cases per 100,000 persons/year in the general population, but increases to 100–200 per 100,000 among individuals over 70 years of age [3]. From a pathophysiological point of view, PD is caused by the progressive degeneration of dopaminergic neurons in the substantia nigra pars compacta (SNc). The resulting dopaminergic dysfunction is responsible for the onset of clinical symptoms [4].

According to the Movement Disorder Society (MDS) Clinical Diagnostic Criteria for PD, the diagnosis of the disease requires bradykinesia (slowness of movement and decreased range of speed during repetitive tasks) in combination with at least one of the following: resting tremor or rigidity [5]. However, the clinical symptoms of PD are not limited to motor impairment but include a wide range of non-motor symptoms (NMS), including neuropsychiatric disorders (e.g., dementia/cognitive impairment, depression and anxiety), autonomic dysfunction (e.g., gastrointestinal problems, orthostatic hypotension, sudomotor alterations), sleep disorders (e.g., REM sleep behavior disorder—RBD), and sensory alterations such as hyposmia [6].

Cognitive decline is a common feature of PD patients and includes all stages of the dementia continuum, ranging from subjective cognitive decline to mild cognitive impairment (MCI) and dementia [2,7,8,9]. In particular, MCI is a relatively early condition, affecting approximately 20% of patients at the time of diagnosis and up to 40–50% after 5 years of follow-up [8,9]. The presence of MCI increases with age and severity of motor impairment, and it is an independent predictor of progression to dementia in individuals with PD [10].

The management of PD typically includes pharmacological and non-pharmacological interventions and, in selected cases, neurosurgery. Given that current treatments are unable to halt neurodegenerative progression, clinical efforts focus on improving symptoms and quality of life [11,12,13]. Pharmacological treatment is based on dopaminergic drugs to compensate for dopamine deficiency. Oral administration of levodopa remains the gold standard for managing motor symptoms [14]. Additional therapeutic options include dopamine agonists, monoamine oxidase B inhibitors, catechol-O-methyltransferase inhibitors, amantadine, and anticholinergics, all aimed at optimizing the effectiveness of levodopa. Among non-pharmacological therapies, particularly in cases complicated by cognitive impairment, non-invasive brain stimulation (NIBS) has shown promising results in recent years. Transcranial magnetic stimulation (TMS) and transcranial direct current stimulation (tDCS) have been explored as adjunct treatments to improve motor and cognitive outcomes in PD [15,16]. TMS, particularly in the form of intermittent theta burst stimulation (iTBS), utilizes patterned high-frequency magnetic pulses to induce electrical currents in the brain, effectively triggering long-term potentiation (LTP)-like plasticity in the targeted cortical areas [17]. In contrast, transcranial electrical stimulation (tES) techniques, such as tDCS, do not reach the action potential activation threshold but instead modulate the resting membrane potential, with anodal stimulation typically increasing cortical excitability [18]. Transcranial random noise stimulation (tRNS) is a unique modality that delivers a random electrical current across a range of frequencies; it is thought to enhance neural signal transmission and processing through stochastic resonance, which may explain the specific improvements observed in processing speed and visual attention in PD-MCI patients [19]. The rationale for targeting the left DLPFC (L-DLPFC) is based on the specific neurobiological and cognitive profile of PD. The L-DLPFC acts as the primary cortical hub within the fronto-striatal circuits [20], which are the most affected by the progressive degeneration of dopaminergic neurons in the substantia nigra. While other frontal regions (e.g., medial frontal, orbitofrontal, ventromedial PFC) are more involved in emotional or motivational processes, the L-DLPFC is essential for executive functioning, working memory, and attentional control, the key domains that define cognitive decline in PD-MCI.

Although episodic memory appears to be a common cognitive target for all modalities, the choice between TMS and tES involves a trade-off between the high focal precision of magnetic stimulation and the portability and potential for prolonged neuromodulation offered by electrical techniques. Unfortunately, the results of NIBS in PD-MCI are still quite heterogeneous due to differences in interventional protocols, which differ in terms of stimulation site, duration, and outcome measures, thus hindering the possibility of drawing definitive conclusions. Therefore, the present systematic review aims to critically evaluate and summarize current evidence on the impact of different NIBS techniques, particularly TMS and tES, on cognitive functions in patients with PD-MCI. By identifying the most effective paradigms and potential response biomarkers, this review seeks to provide clinical insights to optimize non-pharmacological interventions, through NIBS, aimed at modifying cognitive decline in patients with PD and cognitive impairment.

## 2. Materials and Methods

### 2.1. Search Strategy

The research protocol has been registered as a systematic review on the international prospective systematic review registration platform PROSPERO, with registration number CRD420251234982.

As a search strategy, the PubMed, Scopus, Web of Science, and Medline Ultimate (via EBSCO) databases were analyzed for results up to 20 November 2025.

The advanced search query was structured according to specific syntax of each database evaluated and included a combination of keywords including: (1) disease-related terms: “Parkinson’s Disease”, “PD”, “Parkinson”; (2) stimulation-related terms: “NIBS”, “Non Invasive Brain Stimulation”, “TMS”, “Transcranial Magnetic Stimulation”, “tES”, “Transcranial Electrical Stimulation”, “tDCS”, “transcranial Direct Current Stimulation”, “tACS”, “Transcranial Alternating Current Stimulation”, “tRNS”, “Transcranial Random Noise Stimulation”; and (3) cognitive-related terms: “MCI”, “Mild Cognitive Impairment”. The specific search queries for each database can be found in the Supplementary Materials.

### 2.2. Inclusion and Exclusion Criteria

The review involved original articles written in the English language only. Reviews and book chapters were excluded. Paper selection followed the PICO questions: Population, Intervention(s), Comparison(s), Outcome, and Study Design.

#### 2.2.1. Population

The chosen population consisted of PD-MCI patients meeting the following inclusion criteria: adult (≥18 years old), established diagnosis of PD according to MDS clinical diagnostic criteria [5], established diagnosis of MCI according to the Movement Disorder Society (MDS) Task Force guidelines [21], and underwent single or multiple NIBS sessions for the management of cognitive symptoms. The exclusion criteria were: <18 years old, contraindications to NIBS, overt dementia associated with PD (PDD), and neurological comorbidities (other than PD-MCI).

#### 2.2.2. Intervention

The intervention addressed in this review concerned the use of different NIBS techniques, including TMS and tES. Among the former, both single-pulse and repetitive pulses protocols were considered. Regarding tES, tDCS, transcranial alternating current stimulation (tACS), and tRNS were evaluated.

#### 2.2.3. Comparison

Outcome measures of subjects who underwent the stimulation protocol were compared with sham/placebo stimulation or no stimulation group when available, or with pre-intervention values when not available.

#### 2.2.4. Outcome

The outcomes considered in this review included all the cognitive parameters related to MCI in PD patients, namely, neuropsychological tests and scales. To ensure a comprehensive overview, these instruments were categorized into functional domains. For global cognition, instruments such as the Montreal Cognitive Assessment (MoCA) [22] and the Repeatable Battery for the Assessment of Neuropsychological Status (RBANS) [23] were considered prioritary due to their established sensitivity in detecting subtle deficits characteristic of PD-MCI. Regarding memory, episodic components and verbal learning are generally assessed by administering tests such as the Wechsler Memory Scale (WMS) [24] or the Verbal Memory Process Test (VMPT) [25]. Executive function and attention are usually investigated through the frontal assessment battery (FAB) [26], the Stroop Test [27], the Trail Making Test (TMT) [28], and the digit span (forward and backward) [29], which are able to evaluate response inhibition, cognitive flexibility, and working memory. The language domain can be examined using the Boston Naming Test (BNT) [30] and specific verbal fluency tasks. Finally, the assessment of visuospatial skills (including spatial orientation and organization) involves the Judgment of Line Orientation (JLO) [31] and the Rey Complex Figure Test [32]. In addition, the efficacy in terms of cognitive improvement of the different protocols for each NIBS technique was evaluated.

#### 2.2.5. Study Design

Randomized, non-randomized controlled studies and open-label studies assessing the effectiveness of NIBS on cognitive symptoms of PD-MCI patients were included. Conversely, case series and case reports were excluded from the review.

### 2.3. Data Collection, Screening and Data Extraction

#### 2.3.1. Data Collection

Three investigators (A.T., D.M., and A.P.) independently searched relevant information from each study included in the final search output across the four databases (i.e., PubMed, Scopus, Medline Ultimate, and Web of Science). The full search string is available in the Supplementary Materials.

#### 2.3.2. Screening

In the screening phase, after excluding duplicates and non-English-language papers, three investigators (D.M., A.T., and P.A.) independently examined the search output, verifying inclusion and exclusion criteria following different steps. First, titles were examined, then the abstracts, and lastly the full text of the studies. For each step, the investigators provided a response for each study (included/not included).

Any discrepancies between researchers were resolved through discussion; where a consensus could not be reached, a majority vote was used to make the final decision.

#### 2.3.3. Data Extraction

Data were extracted using a pre-designed data extraction template developed specifically for this review, including: study design (e.g., parallel, cross-over), sample size, sex, age, MCI diagnostic level according to the Movement Disorder Society (MDS) Task Force guidelines [21], type of NIBS such as TMS (e.g., repetitive, deep, theta burst) or tES (e.g., tDCS, tACS, tRNS), NIBS protocol details (i.e., stimulation parameters including site of stimulation, intensity, duration, number of sessions/week, and, in case of cross-over design, duration of the washout period), in addition to the cognitive scale results (variation in cognitive results of neuropsychological tests). Three researchers independently extracted data, followed by a cross-check. Any mismatches were resolved by re-examining the original articles.

### 2.4. Risk of Bias Assessment Methodology

The methodological quality of this systematic review was ensured by strict adherence to the AMSTAR 2 (A MeaSurement Tool to Assess systematic Reviews) criteria throughout the review process; furthermore, the study was conducted and reported in accordance with the Preferred Re-porting Items for Systematic Reviews and Meta-Analyses (PRISMA) 2020 statement (both the completed PRISMA 2020 and AMSTAR 2 checklists are provided in the Supplementary Materials).

The risk of bias for the included studies was assessed using the Cochrane Risk of Bias 2 (RoB 2) tool (2019 version) for randomized controlled trials (RCTs) [33]. Specific templates for parallel-group and crossover trials were applied as appropriate. Three researchers (D.M., A.T., and P.A.) independently assessed each study across five domains: (1) randomization process, (2) deviations from intended interventions, (3) missing outcome data, (4) measurement of the outcome, and (5) selection of the reported result. Any disagreements during the assessment process were resolved through discussion or a majority vote. In addition, the ROBINS-I tool for observational studies was used when appropriate. The Grading of Recommendations Assessment, Development and Evaluation (GRADE) approach was used to assess the certainty of the body of evidence for each critical or important outcome. The certainty of evidence was classified into one of four categories: high, moderate, low, or very low. Three researchers independently performed the assessments; any disagreements were resolved through discussion or majority vote.

### 2.5. Data Synthesis

Given the expected methodological heterogeneity among the included studies, particularly regarding stimulation protocols and cognitive assessment tools, a predominantly descriptive synthesis approach was adopted. The descriptive analysis is presented in a series of tables, the first of which focuses on the characteristics of the included studies, while the second concerns the stimulation parameters and the main results of the studies. The primary outcome of interest for this systematic review concerns the results of pre- and post-stimulation neuropsychological tests. The significant findings of each study (expressed as mean ± SD and *p*-value) are discussed in the descriptive analysis. In addition, a narrative synthesis of the findings was conducted.

## 3. Results

### 3.1. Systematic Search Output

**Figure 1:**
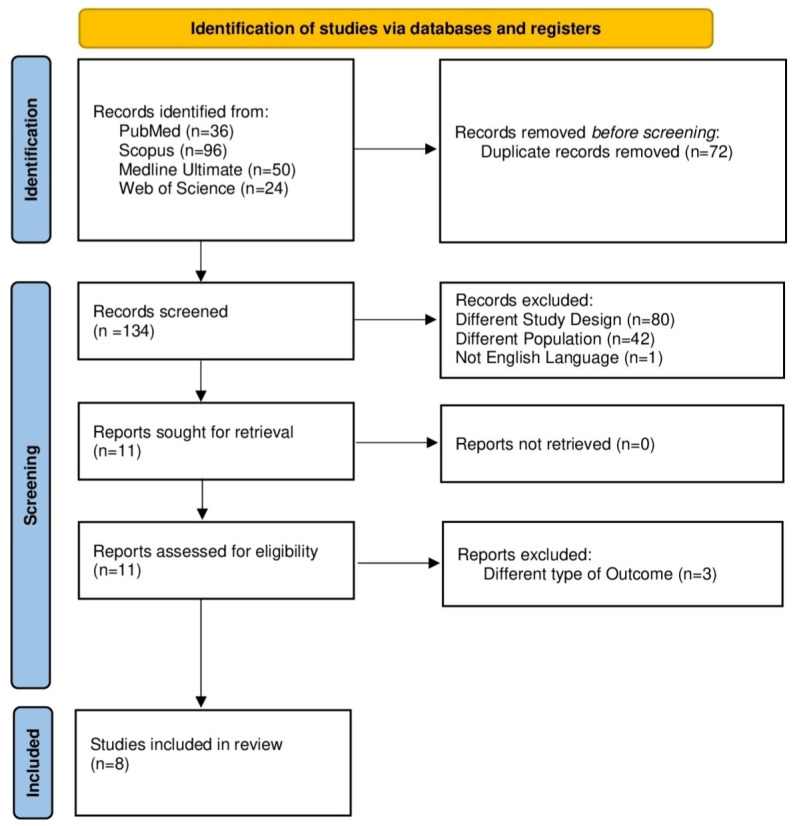
PRISMA flow diagram.

This study systematically searched 4 databases and obtained 206 results distributed with the following search output: PubMed (n = 36), Scopus (n = 96—subject area limited to Medicine, Neuroscience, and Psychology), Medline Ultimate (n = 50—via EBSCO), and Web of Science (n = 24). After removing duplicates (n = 72), according to the inclusion and exclusion criteria, the following studies have been removed due to the following reasons: different study design (n = 80), inappropriate population (n = 42), different type of outcome (n = 3), and non-English language (n = 1) (a complete list of excluded studies with reasons for exclusion is provided in the Supplementary Materials). Thus, following the screening process, 8 studies were ultimately selected based on the inclusion criteria. Figure 1 shows the results using a PRISMA flow diagram.

### 3.2. Study Details

Table 1 reports the characteristics of the included studies, detailing the authors, names and year of publication, study design, sample size, mean age and sex of both experimental and control groups (expressed as mean ± SD), and the diagnostic level of PD-MCI according to the MDS criteria [21].

Table 2 and Table 3 focus on stimulation protocols, highlighting the specific NIBS technique employed, the target site, intensity, duration, and number of stimuli delivered, and the total number of sessions, as well as the outcome measures and the key study findings.

### 3.3. Risk of Bias Assessment Results

**Figure 2:**
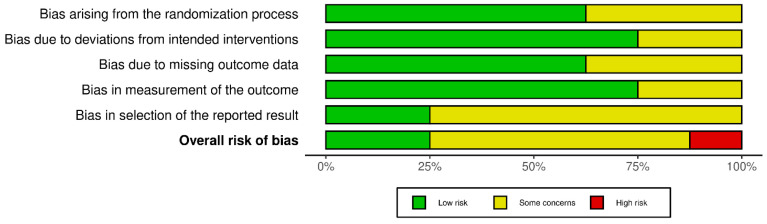
RoB 2 summary plot.

The methodological quality of the included studies was assessed using the Cochrane Risk of Bias 2 (RoB 2) tool. The overall analysis revealed a heterogeneous risk profile among the selected studies (Figure 2).

Only two studies (25%) were classified as having a low overall risk of bias: Lang et al. and Aksu et al. These studies met all the required methodological criteria in every domain. Most studies (n = 5, 62.5%) had “some concerns”: He et al., Lawrence et al., Monastero et al., Cheng et al., and Mazzara et al. The most common issues were the selection of reported outcomes and the lack of details regarding the randomization process. One study (Trung et al.) was classified as having a high risk of bias due to multiple instances of “some concerns” in the domains of outcome measurement and outcome selection, rather than a specific assessment of “High risk” within a single domain.

Regarding the specific domains:

*Domain 1 (Randomization Process)*: The risk was low in most studies (n = 5). However, three studies (Lawrence et al., Monastero et al., Trung et al.) raised “some concerns”, likely due to the lack of a detailed description of random sequence generation or the allocation of concealment.

*Domain 2 (Deviations from Intended Interventions)*: In most cases, the management of protocol deviations was adequate (n = 6 at low risk). Two studies (He et al., Cheng et al.) showed “some concerns,” suggesting potential issues in maintaining blinding or adherence to the assigned protocol.

*Domain 3 (Missing Outcome Data)*: Bias due to data loss (drop-out) was low in five studies. Three studies (He et al., Cheng et al., Trung et al.) were flagged with “some concerns,” indicating that missing data may not have been handled optimally.

*Domain 4 (Measurement of the Outcome)*: Outcome measurement was considered reliable (low risk) in most studies (n = 6). However, the study by Lawrence et al. raised some doubts, while that by Trung et al. received a rating of “some concerns,” probably linked to subjectivity in the administration or evaluation of neuropsychological tests without adequate blinding of outcome assessors.

**Figure 3:**
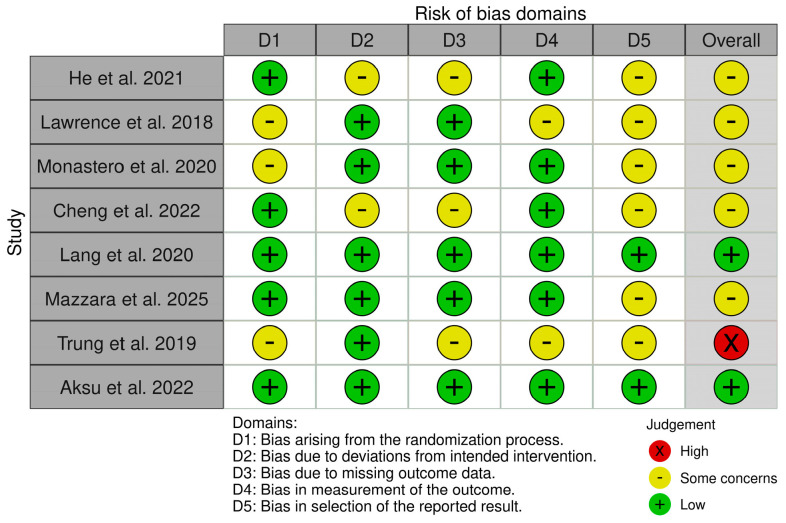
RoB 2 traffic light plot [34,35,36,37,38,39,40,41].

*Domain 5 (Selection of the Reported Result)*: This domain represents the main weakness of the entire review. Only two studies (Lang et al., Aksu et al.) were found to be of low risk, while the remaining six studies showed “some concerns”. Figure 3 presents the single studies’ RoB 2 scores in the form of a traffic light plot.

### 3.4. Descriptive Analysis

Table 1 summarizes the main characteristics of the eight studies included in the systematic review, published between 2018 and 2025. All selected studies adopt RCT design. Most protocols (n = 6) followed a parallel-group design, while two studies (Monastero et al., 2020 and Mazzara et al., 2025) used a crossover design, in which the same patients received both stimulation conditions (real and sham) randomly at different times [36,39].

#### 3.4.1. Population Characteristics

The sample size varies considerably across studies, reflecting the predominantly pilot nature of the various studies. The largest overall sample is found in Lang et al. (2020), with a total of 41 participants (21 in the active group and 20 in the control one) [38], while the smallest sample size is Lawrence et al. (2018), with only 14 total participants (7 per arm) [35]. The average size of treatment groups ranged between 10 and 20 subjects.

The population examined has homogeneous characteristics in terms of age. Regarding gender distribution, almost all studies show a clear male predominance. An extreme case is represented by Monastero et al. (2020), whose sample consisted exclusively of male subjects (M/F = 10/0) [36].

A relevant qualitative issue concerns the diagnostic criteria for MCI: most studies (n = 6, equal to 75%) used a level II diagnosis (comprehensive assessment), which offers greater diagnostic certainty based on in-depth neuropsychological testing; two studies (Monastero et al., 2020 and Mazzara et al., 2025) adopted a level I diagnosis (abbreviated assessment) [36,39].

Overall, the included studies show good homogeneity regarding age and experimental design, but present variability in sample size and a gender bias in favor of the male population, consistent with the epidemiology of PD, which nevertheless requires careful consideration when interpreting the results.

#### 3.4.2. Stimulation Protocols

Table 2 analyzes the technical characteristics of the NIBS interventions used in the eight included studies and the primary findings reported. There is considerable heterogeneity in the adopted techniques, the target sites, and the overall treatment dosage. The included studies are equally divided into two macro-categories of neurostimulation: TMS and tES (n = 4 each). All studies that used TMS (n = 4: Cheng et al., He et al., Lang et al., Trung et al.) adopted intermittent theta burst stimulation (iTBS) [34,37,38,40]. The remaining four studies that used tES employed tDCS (Lawrence et al., Aksu et al.) [35,41] or high-frequency tRNS (Monastero et al., Mazzara et al.) [36,39].

Regarding the stimulation target site, in seven out of eight studies (87.5%), the target chosen was the DLPFC, often specified as the left hemisphere (L-DLPFC), a key area for executive functions; the only exception was the study by Monastero et al., which targeted the motor cortex (MC), primarily testing motor effects but also including secondary cognitive outcomes.

TMS protocols (iTBS/rTMS) are characterized by a very short duration, typically 190 s (or 3 min) per session, with an intensity calibrated to the individual motor threshold (from 80% of AMT to 100% of RMT), while tES protocols (tDCS/tRNS) require longer sessions, ranging between 15 and 20 min. The current intensity was set at 1.5 mA in most cases, except for the study by Aksu et al., which used a higher intensity of 2 mA [41].

The frequency and total number of sessions vary considerably between experimental studies: two studies (Monastero et al., Mazzara et al.) evaluated the acute effects of a single session [36,39], while most studies adopted repeated session protocols, ranging from a minimum of 3 sessions (Trung et al.) [40] to a maximum of 10 sessions (He et al., Cheng et al., Aksu et al.), suggesting a trend towards more intensive treatments to induce long-term plasticity [34,37,41].

#### 3.4.3. Outcome Measures

Analysis of intra-group changes within the active arm reveals a heterogeneous pattern of immediate efficacy, with significant improvements concentrated primarily in episodic memory and global cognition.

The efficacy of NIBS on global cognition varies notably depending on the sensitivity of the assessment tool: studies utilizing MCI-sensitive screening scales, such as the MoCA and RBANS, reported highly statistically significant improvements immediately after treatment, specifically, He et al. (iTBS over L-DLPFC at 100% RMT, 190 s, 10 sessions; MoCA total, pre-treatment: 24.7 ± 2.9, post-treatment: 27.1 ± 2.3, *p* < 0.001; RBANS total, pre-treatment: 88.2 ± 14.7, post-treatment: 96.0 ± 17.6, *p* < 0.001) and Cheng et al. (iTBS over L-DLPFC at 90% RMT, 190 s, 10 sessions; MoCA total, pre-treatment: 23.90 ± 2.92, post-treatment: 26.90 ± 2.18, *p* = 0.005; RBANS, pre-treatment: 84.36 ± 13.95, post-treatment: 90.55 ± 15.76, *p* = 0.005) [34,37]. Mazzara et al. (tRNS over L-DLPFC at 1.5 mA for 15 min, 1 session) confirmed this finding with a significant improvement in the total MoCA score (pre-treatment: 22.0 ± 3.8, post-treatment: 23.9 ± 4.2, *p* = 0.018) [39].

Memory appears to be the cognitive domain responding most consistently and robustly to short-term stimulation. Strongly significant results emerged in Aksu et al. (tDCS, L-DLPFC, 2 mA, 20 min, 10 sessions) for the Wechsler Memory Scale (WMS) Logical Memory immediate (pre-treatment: 13.92 ± 0.27, post-treatment: 17.92 ± 3.79, *p* = 0.0024); WMS Logical Memory delayed (pre-treatment: 16.77 ± 4.41, post-treatment: 20.38 ± 4.07, *p* < 0.001) and Verbal Memory Process Test (VMPT) total score (pre-treatment: 14.61 ± 0.76, post-treatment: 14.61 ± 0.86, *p* < 0.001) [41]. Similarly, He et al. (RBANS immediate memory, pre-treatment: 92.5 ± 19.7, post-treatment: 100.8 ± 19.7, *p* = 0.001; RBANS delayed memory, pre-treatment: 96.1 ± 17.9, post-treatment: 102.2 ± 21.4, *p* = 0.001; MoCA delayed recall, pre-treatment: 2.2 ± 1.6, post-treatment: 3.6 ± 1.0, *p* = 0.001) and Cheng et al. (RBANS immediate memory, pre-treatment: 85.82 ± 17.19, post-treatment: 94.36 ± 20.89, *p* = 0.016; RBANS delayed memory, pre-treatment: 94.73 ± 21.90, post-treatment: 99.45 ± 25.62, *p* = 0.018; MoCA delayed recall, pre-treatment: 1.80 ± 1.69, post-treatment: 3.20 ± 1.23, *p* = 0.011) report significant improvements in both immediate and delayed memory in the RBANS and in MoCA delayed recall [34,37].

Regarding working memory, results are less uniform. While He et al. (RBANS Attention, pre-treatment: 94.2 ± 16.0, post-treatment: 96.6 ± 16.3, *p* = 0.068) and Cheng et al. (RBANS Attention, pre-treatment: 90.18 ± 12.40, post-treatment: 91.82 ± 14.02, *p* = 0.345) did not detect significant improvements in the RBANS “Attention” domain, other studies showed no effects on the digit span: Monastero et al. (tRNS protocol, L-MC, 1.5 mA, 15 min, 1 session) (digit span forward, pre-treatment: 3.6 ± 0.5, post- treatment: 3.4 ± 0.9, *p* = 0.500; digit span backward, pre-treatment: 2.5 ± 0.7; post-treatment: 2.5 ± 0.7, *p* = 0.433), Aksu et al. (digit span forward, pre-treatment: 4.30 ± 1.10, post-treatment: 4.92 ± 1.32, *p* = 0.128; digit span backward, pre-treatment: 3.38 ± 1.04, post-treatment: 3.15 ± 0.89, *p* = 0.621), and Trung et al. (iTBS protocol, L-DLPFC, 80% AMT, 190 s, 3 sessions) (digit span forward and digit span backward in z-scores, pre-treatment: −0.504, post-treatment: −0.446, *p* = ns) [34,36,37,40,41].

The effects on executive functions are heterogeneous and depend on the specific subdomain tested. Mazzara et al. report a significant improvement in the Frontal Assessment Battery (FAB) (pre-treatment: 14.7 ± 2.6, post-treatment: 15.7 ± 2.7, *p* = 0.022) [39]. However, this finding was not replicated in Monastero et al. (Stroop Test Errors, pre-treatment: 3.7 ± 5.3, post-treatment: 3.9 ± 4.1, *p* = 0.722; Stroop Test Time, pre-treatment: 43.1 ± 20.9, post-treatment: 40.7 ± 30.2, *p* = 0.631) or Aksu et al. (Stroop Interference Time, pre-treatment: 79.53 ± 51.1, post-treatment: 59.76 ± 46.5, *p* = 0.243) [36,41].

Regarding processing speed and visual attention, Monastero et al. highlight significant improvements in the Digit Symbol (pre-treatment: 17.1 ± 13.5, post-treatment: 20.4 ± 15.6, *p* = 0.019) and, in particular, in the Visual Search (pre-treatment: 34.5 ± 10.2, post-treatment: 42.1 ± 9.9, *p* < 0.0001) [36]. It should be noted that in a study by Lang et al. (iTBS protocol, L-DLPFC, 80% AMT, 3 min, 6 sessions) focusing on executive functions, no significant improvements were detected in the immediate post-test (Stroop Color and Word Test, Brixton Spatial Anticipation Hayling Sentence Completion Section 2, Trail Making Test B, clock-drawing test command in z-scores, pre-treatment: −0.87 ± 0.66, post-treatment: −0.47 ± 0.65, *p* = ns) [38].

Results relating to language showed discrepancies among studies. Aksu et al. reported a highly significant improvement in the Boston Naming Test (BNT, pre-treatment: 20.75 ± 4.49, post-treatment: 22.75 ± 4.28, *p* < 0.001) [41]. Conversely, Lang et al. (BNT in z-scores, pre-treatment: −0.47 ± 0.83, post-treatment: −0.46 ± 0.75, *p* = ns) using the same test did not show significant variations [38]. Specific language subdomains (measured via MoCA or RBANS) appear improved in He et al. (MoCA Language, pre-treatment: 2.0 ± 0.5, post-treatment: 2.4 ± 0.6, *p* = 0.013) and Cheng et al. (RBANS Language, pre-treatment: 90.27 ± 14.16, post-treatment: 94.55 ± 15.71, *p* = 0.038), while verbal fluency (FAS/COWAT) tends not to reach statistical significance in the short term, although Mazzara et al. report a positive trend (FAS, pre-treatment: 28.4 ± 12.0, post-treatment: 31.0 ± 12.5, *p* = 0.087) [34,37,39].

Lastly, visuospatial skills appear to be less responsive in the short term, with notable exceptions. Trung et al. identify visuospatial skills as the only significantly improved domain immediately post-test (Hooper Visual Organization Test, Rey Complex Figure Test, clock-drawing test composite score: pre-treatment: 0.596, post-treatment: 0.116, *p* = 0.003) [40]. He et al. reported improvement in the visuospatial domain of the RBANS (RBANS Visuospatial, pre-treatment: 89.9 ± 15.4, post-treatment: 95.7 ± 18.1, *p* = 0.019) [34]. Most other studies, such as Cheng et al. (MoCA Orientation, pre-treatment: 5.60 ± 0.97, post-treatment: 5.60 ± 0.97, *p* = 1000), Lang et al. (JLO and Rey Complex Figure Test, pre-treatment: −0.72 ± 0.81, post-treatment: −0.75 ± 0.78, *p* = ns), and Aksu et al. (JLO, pre-treatment: 15.23 ± 6.23, post-treatment: 16.23 ± 5.80, *p* = 0.304), reported no significant changes (*p* > 0.05) [37,38,41].

The direct comparison between active and sham stimulation (Table 3) reveals the statistical superiority of active NIBS in only a subset of the included studies, with more consistent results observed in the domains of global cognition and episodic memory. Active stimulation yielded significantly greater improvements compared to sham in the total MoCA score in He et al. (*p* < 0.001) and Mazzara et al. (*p* = 0.017), and in the RBANS total score in Cheng et al. (*p* = 0.014) [34,37,39]. Significant results in favor of the active group also emerged for long-term memory (Paragraph Recall, *p* = 0.001 in Lawrence et al. [35] (tDCS over L-DLPFC at 1.5 mA for 20 min, 4 sessions); MoCA Recall, *p* = 0.018 in Mazzara et al. [39] and for certain executive functions (Stroop Test, *p* = 0.04 in Lawrence et al.) [35]; FAB, *p* = 0.001 in Mazzara et al. [39]. However, it is important to note that in the studies by Monastero et al., Lang et al., and Trung et al., the between-groups comparisons did not reach statistical significance for any of the cognitive parameters assessed (*p* > 0.05), indicating comparable effects between active and sham stimulation in these specific protocols [36,38,40].

## 4. Discussion

This systematic review evaluated the efficacy of NIBS techniques on cognitive deficits in patients with PD-MCI. The qualitative analysis of the eight included RCTs suggests that, although NIBS (both TMS and tES) shows promising therapeutic potential, its effectiveness varies significantly depending on the cognitive domain assessed, the stimulation protocol, and the sensitivity of the psychometric tools used.

The most consistent finding from the analysis concerns memory and global cognition. Specifically, our results indicate that episodic memory is the most responsive domain, suggesting that modulation of the DLPFC may facilitate impaired hippocampal–frontal retrieval circuits in PD. However, the detection of these improvements is highly dependent on instrumental sensitivity. Studies that used sensitive tests for MCI, such as RBANS and MoCA, reported significant improvements compared to placebo. Conversely, studies using tests of global cognition such as the MMSE have failed to detect changes in cognitive functioning that emerge instead with more sensitive tools [35]. This is consistent with a recent network meta-analysis by Fan et al. (2025), which identifies variability of measurement instruments as a critical confounding factor [42]. The choice of assessment tools is indeed a critical factor in evaluating NIBS efficacy. The strengths of the current evidence include the use of MCI-specific scales like the MoCA and RBANS, which demonstrate superior sensitivity in detecting subtle cognitive changes induced by neurostimulation in PD-MCI compared to general screening tools such as the MMSE. Furthermore, domain-specific tests (i.e., WMS for memory or FAB for executive functions) allow for the identification of specific neuroplastic changes related to the stimulation target, such as the L-DLPFC.

However, certain weaknesses must be acknowledged: a primary limitation is the susceptibility of many tests, particularly those measuring global cognition, to practice effects, where increased scores in follow-up assessments may reflect learning rather than actual benefits of the intervention. Furthermore, the continued use of less sensitive instruments such as the MMSE in some studies often fails to detect improvements that are captured by more extensive Level II diagnostic cognitive batteries.

The analysis of the comparison between active vs. sham treatments highlights the difficulty of isolating the pure therapeutic effect of NIBS in a complex population such as PD-MCI, in which a potential placebo or learning effect (practice effect) may mask differences between groups. The lack of intergroup significance found in several studies (e.g., Lang et al., Trung et al.) [38,40] suggests that the improvement observed in the active group may not always be robust enough to statistically overcome the variation in the sham group in the short term. This phenomenon appears to be influenced, again, by the sensitivity of the measurement tools, but, in addition, the superiority of active NIBS appears to be even related to the stimulation volume, with more favorable inter-group results in protocols involving repeated sessions compared to those using a single session. Moreover, another variable is the heterogeneity of results among studies regarding “monotherapy” versus those combining NIBS with cognitive training. This aspect finds fundamental support when compared with Fan et al. (2025), who demonstrated that the combination of NIBS and cognitive rehabilitation (CR) represents the most effective approach for cognitive improvement [42]. As hypothesized by these authors, NIBS may act by modulating cortical excitability and inducing LTP-like plasticity, creating a “primed” neuronal substrate that is effectively shaped by concomitant cognitive exercise [42].

In contrast, studies in our review that did not include a concomitant structured cognitive task showed more variable effects. For instance, Lang et al. (iTBS alone) found no immediate improvements in executive functions [38]. Similarly, Monastero et al. found no executive changes, although this may also be attributable to the fact that the target stimulation was MC rather than the DLPFC [36]. This suggests that both the specific target (i.e., DLPFC) and the concurrent activity (i.e., CR) are critical for maximizing executive outcomes.

Finally, a critical issue concerns the timing of assessment. Most studies have focused on immediate effects. However, as highlighted by Lang et al. (2020), the absence of immediate improvements does not preclude long-term benefits [38]. In their study, executive improvements emerged only after a month of follow-up, suggesting that neuroplastic mechanisms may require a consolidation phase to translate into detectable behavioral gains. Conversely, studies such as Trung et al. (2019) show immediate improvements (e.g., visuospatial skills) that tend to be maintained [40].

The present systematic review has several strengths, such as its methodical approach, particularly with regard to the study population (confirmed PD-MCI patients), and the rigorous selection of studies with clear and reproducible NIBS interventions. However, this review shows certain limitations. First, the small sample size of the included studies (often < 20 patients per arm) reduces their statistical power significantly. Second, the heterogeneity of the outcome measures, protocols (iTBS vs. tDCS vs. tRNS), and stimulation sites (DLPFC vs. M1) hinders a direct comparison of the different techniques and, consequently, the possibility of conducting a meta-analysis. Furthermore, the short-term follow-up limits any conclusions on long-term effects of NIBS in such patients.

## 5. Conclusions

NIBS represents an adjunctive emerging therapeutic tool for treating PD-MCI; although its clinical utility remains to be fully established through larger scale clinical trials. However, there is preliminary but consistent evidence supporting improvement in memory and global cognition after NIBS treatment in PD-MCI patients. However, its effectiveness appears to be closely dependent on the chosen target of stimulation, its intensity, duration, and number of sessions. Most studies have focused on immediate effects, while future research should prioritize combined protocols (NIBS + CR) and include medium-term follow-up assessments (1–3 months) to capture delayed effects of neurostimulation on neuroplasticity. Most of the studies included in this review present small sample sizes, heterogeneous protocols, and short-term follow-up, which limits generalizability of results. Regarding the cognitive domains, results about executive function and attention outcomes show elevated variability, while memory and global cognition show more reliable improvements, even if the overall certainty of evidence is low to moderate.

Prospective studies conducted on larger cohorts of subjects evaluated with extensive neuropsychological batteries (i.e., level II MDS diagnostic criteria for PD-MCI) will be necessary to confirm the best stimulation technique and optimal stimulation parameters to use for cognitive impairment in nondemented individuals with PD.

## Figures and Tables

**Table 1:** Demographic characteristics, study design, and PD-MCI MDS diagnostic level of the included studies.

Author and Year	Study Design	Sample Size Active/Control	Age (Mean ± SD) Active/Control	Sex (M/F) Active–Control	MCI Diagnostic Level
He et al., 2021 [34]	RCT	20/15	70.0 ± 6.3/74.8 ± 6.9	13/7–10/5	II
Lawrence et al., 2018 [35]	RCT	7/7	72.00 ± 6.45/72.29 ± 6.21	5/2–4/3	II
Monastero et al., 2020 [36]	RCT (Cross-Over)	10/10	70.6 ± 7.8/70.6 ± 7.8	10/0–10/0	I
Cheng et al., 2022 [37]	RCT	11/16	71.6 ± 5.1/73.9 ± 6.9	6/5–11/5	II
Lang et al., 2020 [38]	RCT	21/20	68.43 ± 8.4/68.76 ± 8.3	14/7–13/7	II
Mazzara et al., 2025 [39]	RCT (Cross-Over)	10/10	67.0 ± 7.4/67.0 ± 7.4	8/2–8/2	I
Trung et al., 2019 [40]	RCT	14/14	71.3 ± 7.3/67.3 ± 5.2	8/6–11/3	II
Aksu et al., 2022 [41]	RCT	13/13	65.52 ± 7.49	17/09	II

**Table 2:** Stimulation protocols and significant results (pre- vs. post-stimulation *p*-value of the active group) of the included studies.

Authors and Year	NIBS Technique	Target	Intensity	Duration/Number of Sessions	Outcome Measures	Main Improvements (Pre- vs. Post-Stimulation *p*-Value of the Active Group)
He et al., 2021 [34]	iTBS	L-DLPFC	100% RMT	190 s/10	RBANS Total	<0.001
					RBANS-IM	0.001
					RBANS-DM	0.001
					RBANS Visuospatial	0.019
					MoCA Total	<0.001
					MoCA Language	0.013
					MoCA Recall	0.001
Monastero et al., 2020 [36]	tRNS	L-MC	1.5 mA 100–600 Hz	15 min/1	Digit Symbol	0.019
					Visual Search	<0.0001
Cheng et al., 2022 [37]	iTBS	L-DLPFC	90% RMT	190 s (600 pulses)/10	RBANS Total	0.005
					RBANS-IM	0.016
					RBANS-DM	0.018
					RBANS Language	0.038
					MoCA Total	0.005
					MoCA Language	0.020
					MoCA Recall	0.011
Lang et al. 2020 [38]	iTBS	L-DLPFC	80% AMT	3 min (600 pulses)/6	Global Cognition	>0.05 *
					Executive Functions	>0.05 *
					Language	>0.05 *
					Attention	>0.05 *
					Memory	>0.05 *
					Visuospatial	>0.05 *
Mazzara et al., 2025 [39]	tRNS	L-DLPFC	1.5 mA 100–600 Hz	15 min/1	MoCA Total	0.018
					FAB	0.022
Trung et al., 2019 [40]	iTBS	L-DLPFC	80% AMT	190 s/3	Visuospatial abilities	0.03
Aksu et al., 2022 [41]	tDCS	L-DLPFC	2 mA	20 min/10	Boston Naming Test	<0.001
					VMPT Total	<0.001
					WMS Logical Memory (Immediate)	0.0024
					WMS Logical Mem (Delayed)	<0.001

Abbreviations: AMT = active motor threshold; FAB = frontal assessment battery; iTBS = intermittent theta burst stimulation; L-DLPFC = left dorsolateral prefrontal cortex; L-MC = Left Motor Cortex; MoCA = Montreal Cognitive Assessment; *p* > 0.05 * = no significant improvement; RBANS = Repeatable Battery for the Assessment of Neuropsychological Status; RBANS-DM = delayed memory; RBANS-IM = immediate memory; RMT = resting motor threshold; tDCS = transcranial direct current stimulation; tRNS = transcranial random noise stimulation; VMPT = Verbal Memory Process Test; WMS = Wechsler Memory Scale.

**Table 3:** Stimulation protocols and significant results (active vs. sham *p*-value) of the included studies.

Authors and Year	NIBS Technique	Target	Intensity	Duration/Number of Sessions	Outcome Measures	Main Improvements (Active vs. Sham *p*-Value)
He et al., 2021 [34]	iTBS	L-DLPFC	100% RMT	190 s/10	RBANS Total	<0.001
					RBANS-IM	0.001
					RBANS-DM	0.017
					MoCA Total	<0.001
					MoCA Language	0.004
					MoCA Recall	0.006
Lawrence et al., 2018 [35]	tDCS	L-DLPFC	1.5 mA	20 min/4	Stroop Test	0.04
					Paragraph Recall	0.001
Monastero et al., 2020 [36]	tRNS	L-MC	1.5 mA 100–600 Hz	15 min/1	Digit Symbol	>0.05 *
					Digit Span Forward	>0.05 *
					Digit Span Backward	>0.05 *
					Visual Search	>0.05 *
					Stroop Errors	>0.05 *
					Stroop Time	>0.05 *
					Letter Fluency	>0.05 *
Cheng et al., 2022 [37]	iTBS	L-DLPFC	90% RMT	190 s (600 pulses)/10	RBANS Total	0.014
					MoCA Language	0.024
Lang et al., 2020 [38]	iTBS	L-DLPFC	80% AMT	3 min (600 pulses)/6	Global Cognition	>0.05 *
					Executive Functions	>0.05 *
					Language	>0.05 *
					Attention	>0.05 *
					Memory	>0.05 *
					Visuospatial	>0.05 *
Mazzara et al., 2025 [39]	tRNS	L-DLPFC	1.5 mA 100–600 Hz	15 min/1	MoCA Total	0.017
					MoCA Recall	0.018
					FAB	0.001
Trung et al., 2019 [40]	iTBS	L-DLPFC	80% AMT	190 s/3	Attention and Working Memory	>0.05 *
					Executive functions	>0.05 *
					Language	>0.05 *
					Memory and Verbal Learning	>0.05 *
					Visuospatial Abilities	>0.05 *
					MoCA	>0.05 *
Aksu et al., 2022 [41]	tDCS	L-DLPFC	2 mA	20 min/10	Digit Span Forward	NR
					Digit Span Backward	NR
					TMT A	NR
					Stroop Interference Time	NR
					Semantic Fluency	NR
					COWAT	NR
					Boston Naming Test	NR
					JLO	NR
					BFRT	NR
					VMPT	NR
					WMS Logical Mem (Immediate)	NR
					WMS Logical Mem (Delayed)	NR

Abbreviations: AMT = active motor threshold; BFRT = Benton Facial Recognition Test; COWAT = Controlled Oral Word Association Test; FAB = frontal assessment battery; iTBS = intermittent theta burst stimulation; JLO = Judgment of Line Orientation; L-DLPFC = left dorsolateral prefrontal cortex; L-MC = Left Motor Cortex; MoCA = Montreal Cognitive Assessment; NR = Not Reported; *p* > 0.05 * = no significant improvement; RBANS = Repeatable Battery for the Assessment of Neuropsychological Status; RBANS-DM = delayed memory; RBANS-IM = immediate memory; RMT = resting motor threshold; tDCS = transcranial direct current stimulation; TMT A = Trail Making Test part A; tRNS = transcranial random noise stimulation; VMPT = Verbal Memory Process Test; WMS = Wechsler Memory Scale.

## Data Availability

No new data were created or analyzed in this study. Data sharing is not applicable to this article.

## Supplementary Materials

The following supporting information can be downloaded at https://www.mdpi.com/article/10.3390/brainsci16030325/s1: File S1: AMSTAR-Assessing the Methodological Quality of Systematic Reviews, File S2: List of excluded studies with motivation, File S3: PRISMA Checklist, File S4: Systematic review databases search queries. Reference [43] is cited in the supplementary materials.

## Abbreviations

The following abbreviations are used in this manuscript:

AMT	Active motor threshold
AMSTAR	A MeaSurement Tool to Assess systematic Reviews
DLPFC	Dorsolateral prefrontal cortex
FAB	Frontal assessment battery
iTBS	Intermittent theta burst stimulation
MC	Motor cortex
MCI	Mild cognitive impairment
MMSE	Mini-Mental State Examination test
MoCA	Montreal Cognitive Assessment
PD	Parkinson’s disease
PD-MCI	Parkinson’s disease—mild cognitive impairment
RBANS	Repeatable Battery for the Assessment of Neuropsychological Status
RMT	Resting motor threshold
rTMS	Repetitive transcranial magnetic stimulation
RoB2	Risk of bias
tACS	Transcranial alternating current stimulation
tDCS	Transcranial direct current stimulation
tES	Transcranial electrical stimulation
TMS	Transcranial magnetic stimulation
tRNS	Transcranial random noise stimulation
VMT	Verbal Memory Process Test
WMS	Wechsler Memory Scale
