# Supplementary material for: The Use of Non-Invasive Brain Stimulation Techniques in Subjects with Parkinson’s Disease and Mild Cognitive Impairment: A Systematic Review
Source: Brain Sci. 2026 Mar 19;16(3):325. doi: 10.3390/brainsci16030325 (PMC13024718; doi:10.3390/brainsci16030325)
Supplement: Supplementary file 1 [file brainsci-16-00325-s001.zip › File S2. list of excluded studies with motivation.pdf]

Study	Reason for exclusion
Devi G. A how-to guide for a precision medicine approach to the diagnosis and treatment of Alzheimer's disease. Front Aging Neurosci. 2023 Aug 17;15:1213968. doi: 10.3389/fnagi.2023.1213968.	Different type of study (opinion Paper)
Inagawa T, Narita Z, Sugawara N, Maruo K, Stickley A, Yokoi Y, Sumiyoshi T. A Meta-Analysis of the Effect of Multisession Transcranial Direct Current Stimulation on Cognition in Dementia and Mild Cognitive Impairment. Clin EEG Neurosci. 2019 Jul;50(4):273-282. doi: 10.1177/1550059418800889. Epub 2018 Sep 19.	Different type of study (Meta-Analysis)
Aghamoosa S, Lopez J, Rbeiz K, Fleischmann HH, Horn O, Madden K, Caulfield KA, Antonucci MU, Revuelta G, McTeague LM, Benitez A. A phase I trial of accelerated intermittent theta burst rTMS for amnestic MCI. J Neurol Neurosurg Psychiatry. 2024 Oct 16;95(11):1036-1045. doi: 10.1136/jnnp-2023-332680. PMID: 38719432.	Different type of Population
Zeyu Y, Liang Z, Jia W, Wang J, Zha Q, Yulong W, Jianjun L. A visualized analysis of research hotspots in high-frequency repetitive transcranial magnetic stimulation from the macroscopic perspective. Chin J Tissue Eng Res. 2026;30(5):1320-1330. doi: 10.12307/2026.033.	Different type of study (Review)
Lane HY, Wang SH, Lin CH. Adjunctive transcranial direct current stimulation (tDCS) plus sodium benzoate for the treatment of early-phase Alzheimer's disease: A randomized, double-blind, placebo-controlled trial. Psychiatry Res. 2023 Oct;328:115461. doi: 10.1016/j.psychres.2023.115461. Epub 2023 Sep 7. PMID: 37729717.	Different type of Population
Godefroy V, Sezer I, Bouzigues A, Montembeault M, Koban L, Plassmann H, Migliaccio R. Altered delay discounting in neurodegeneration: insight into the underlying mechanisms and perspectives for clinical applications. Neurosci Biobehav Rev. 2023 Mar;146:105048. doi: 10.1016/j.neubiorev.2023.105048. Epub 2023 Jan 18.	Different type of study (Review)
Liu H, Wang XP. Alternative Therapies for Non-Motor Symptoms in Parkinson's Disease: A Mini Review. Neuropsychiatr Dis Treat. 2024 Dec 21;20:2585-2591. doi: 10.2147/NDT.S495092. PMID: 39723118.	Different type of study (Review)
Schirinzi T, Di Lorenzo F, Sancesario GM, Di Lazzaro G, Ponzo V, Pisani A, Mercuri NB, Koch G, Martorana A.	Different type of Population

Amyloid-Mediated Cholinergic Dysfunction in Motor Impairment Related to Alzheimer's Disease. J Alzheimers Dis. 2018;64(2):525-532. doi: 10.3233/JAD-171166.	
Pagonabarraga J, Kulisevsky J. Apathy in Parkinson's Disease. Int Rev Neurobiol. 2017;133:657-678. doi: 10.1016/bs.irn.2017.05.025. Epub 2017 Jul 10.	Different type of study (Review)
Zhang Q, Aldridge GM, Narayanan NS, Anderson SW, Uc EY. Approach to Cognitive Impairment in Parkinson's Disease. Neurotherapeutics. 2020 Oct;17(4):1495-1510. doi: 10.1007/s13311-020-00963-x. Epub 2020 Nov 17.	Different type of study (Review)
Vaccaro MG, Pullano L, Canino S, Pastore M, Sarica A, Quattrone A, Fernandes SM, Migliorini F, Maestu F, Quattrone A. Assessing of the Italian version of the Memory Strategy Test (TMS) in people with Parkinson disease: a preliminary descriptive psychometric study. Neurol Sci. 2023 Nov;44(11):3895-3903. doi: 10.1007/s10072-023-06906-6. Epub 2023 Jun 24.	different type of study (Test Validation)
Trojsi F, Christidi F, Migliaccio R, Santamaría-García H, Santangelo G. Behavioural and Cognitive Changes in Neurodegenerative Diseases and Brain Injury. Behav Neurol. 2018 Jul 25;2018:4935915. doi: 10.1155/2018/4935915.	Different type of study (Review)
Jun P, Chengye H, Hui W. Bibliometric analysis of rehabilitation in Alzheimer's disease (2000-2023): trends, hotspots and prospects. Front Aging Neurosci. 2024 Dec 3;16:1457982. doi: 10.3389/fnagi.2024.1457982. PMID: 39691162.	Different type of study (Bibliometric Analysis)
Song BX, Vieira E, Gallagher D, Diniz BS, Fischer CE, Flint AJ, Herrmann N, Mah L, Mulsant BH, Rajji TK, Ma C, Lanctôt KL; PACt-MD Study Group. Blood Angiogenesis Markers and Cognition in Older Adults at Risk for Dementia: Marqueurs sanguins de l'angiogenèse et cognition chez les personnes âgées à risque de démence. Can J Psychiatry. 2025 Apr 30:7067437251337627. doi: 10.1177/07067437251337627. Epub ahead of print.	Different type of Population
Miraglia F, Vecchio F, Pappalettera C, Nucci L, Cotelli M, Judica E, Ferreri F, Rossini PM. Brain Connectivity and Graph Theory Analysis in Alzheimer's and Parkinson's Disease: The Contribution of Electrophysiological Techniques. Brain Sci. 2022 Mar 18;12(3):402. doi: 10.3390/brainsci12030402.	Different type of study (Review)

Moshayedi AJ, Mokhtari T, Emadi Andani M. Brain Stimulation Techniques in Research and Clinical Practice: A Comprehensive Review of Applications and Therapeutic Potential in Parkinson's Disease. Brain Sci. 2024 Dec 27;15(1):20. doi: 10.3390/brainsci15010020.	Different type of Study (Review)
Burdette D, Mirro EA, Lawrence M, Patra SE. Brain-responsive corticothalamic stimulation in the pulvinar nucleus for the treatment of regional neocortical epilepsy: A case series. Epilepsia Open. 2021 Sep;6(3):611-617. doi: 10.1002/epi4.12524. Epub 2021 Aug 3.	Different type of Population
Saha C, Figley CR, Lithgow B, Fitzgerald PB, Koski L, Mansouri B, Anssari N, Wang X, Moussavi Z. Can Brain Volume-Driven Characteristic Features Predict the Response of Alzheimer's Patients to Repetitive Transcranial Magnetic Stimulation? A Pilot Study. Brain Sci. 2024 Feb 28;14(3):226. doi: 10.3390/brainsci14030226. PMID: 38539615; PMCID: PMC10968477.	Different type of Population
de la Torre JC, Olmo AD, Valles S. Can mild cognitive impairment be stabilized by showering brain mitochondria with laser photons? Neuropharmacology. 2020 Jul;171:107841. doi: 10.1016/j.neuropharm.2019.107841. Epub 2019 Nov 5.	Different type of Population
Reumers SFI, Maas RPPWM, Schutter DJLG, Teerenstra S, Kessels RPC, de Leeuw FE, van de Warrenburg BPC. Cerebellar Transcranial Direct Current Stimulation in the Cerebellar Cognitive Affective Syndrome: A Randomized, Double-Blind, Sham-Controlled Trial. Mov Disord. 2025 Jan;40(1):121-131. doi: 10.1002/mds.30043. Epub 2024 Nov 2.	Different type of Population
Tadayon E, Pascual-Leone A, Press D, Santarnecchi E; Alzheimer's Disease Neuroimaging Initiative. Choroid plexus volume is associated with levels of CSF proteins: relevance for Alzheimer's and Parkinson's disease. Neurobiol Aging. 2020 May;89:108-117. doi: 10.1016/j.neurobiolaging.2020.01.005. Epub 2020 Jan 16.	Different type of Population
Marcos-Frutos D, López-Alonso V, Mera-González I, Sánchez-Molina JA, Colomer-Poveda D, Márquez G. Chronic Functional Adaptations Induced by the Application of Transcranial Direct Current Stimulation Combined with Exercise Programs: A Systematic Review of Randomized	Different type of study (Review)

Controlled Trials. J Clin Med. 2023 Oct 24;12(21):6724. doi: 10.3390/jcm12216724.	
Shen Y, Lv QK, Xie WY, Gong SY, Zhuang S, Liu JY, Mao CJ, Liu CF. Circadian disruption and sleep disorders in neurodegeneration. Transl Neurodegener. 2023 Feb 13;12(1):8. doi: 10.1186/s40035-023-00340-6.	Different type of study (Review)
Safarpour D, Willis AW. Clinical Epidemiology, Evaluation, and Management of Dementia in Parkinson Disease. Am J Alzheimers Dis Other Demen. 2016 Nov;31(7):585-594. doi: 10.1177/1533317516653823. Epub 2016 Jun 13. PMID: 27295974.	Different type of study (Review)
Kim YE, Jeon BS. Clinical implication of REM sleep behavior disorder in Parkinson's disease. J Parkinsons Dis. 2014;4(2):237-44. doi: 10.3233/JPD-130293.	Different type of study (Review)
Tikka SK, Siddiqui MA, Garg S, Pattojoshi A, Gautam M. Clinical Practice Guidelines for the Therapeutic Use of Repetitive Transcranial Magnetic Stimulation in Neuropsychiatric Disorders. Indian J Psychiatry. 2023 Feb;65(2):270-288. doi: 10.4103/indianjpsychiatry.indianjpsychiatry_492_22. Epub 2023 Jan 30.	Different type of study (Clinic Guideline)
O'Brien JT, Holmes C, Jones M, Jones R, Livingston G, McKeith I, Mittler P, Passmore P, Ritchie C, Robinson L, Sampson EL, Taylor JP, Thomas A, Burns A. Clinical practice with anti-dementia drugs: A revised (third) consensus statement from the British Association for Psychopharmacology. J Psychopharmacol. 2017 Feb;31(2):147-168. doi: 10.1177/0269881116680924. Epub 2017 Jan 20.	Different type of study (Consensus Statement)
Murugaraja V, Shivakumar V, Sivakumar PT, Sinha P, Venkatasubramanian G. Clinical utility and tolerability of transcranial direct current stimulation in mild cognitive impairment. Asian J Psychiatr. 2017 Dec;30:135-140. doi: 10.1016/j.ajp.2017.09.001. Epub 2017 Sep 8. PMID: 28934620.	Different type of Population
Anderkova L, Rektorova I. Cognitive effects of repetitive transcranial magnetic stimulation in patients with neurodegenerative diseases - clinician's perspective. J Neurol Sci. 2014 Apr 15;339(1-2):15-25. doi: 10.1016/j.jns.2014.01.037. Epub 2014 Feb 4.	Different type of Study (Review)

Chen H, Wang X, Zhang J, Xie D. Effect of high-frequency repetitive transcranial magnetic stimulation on cognitive impairment in WD patients based on inverse probability weighting of propensity scores. Front Neurosci. 2024 Apr 10;18:1375234. doi: 10.3389/fnins.2024.1375234.	Different type of Population
Krellman JW, Mercuri G. Cognitive Interventions for Neurodegenerative Disease. Curr Neurol Neurosci Rep. 2023 Sep;23(9):461-468. doi: 10.1007/s11910-023-01283-1. Epub 2023 Jul 10.	Different type of study (Review)
Celebi O, Temuçin ÇM, Elibol B, Saka E. Cognitive profiling in relation to short latency afferent inhibition of frontal cortex in multiple system atrophy. Parkinsonism Relat Disord. 2014 Jun;20(6):632-6. doi: 10.1016/j.parkreldis.2014.03.012. Epub 2014 Mar 19.	Different type of Population
Biundo R, Weis L, Fiorenzato E, Antonini A. Cognitive Rehabilitation in Parkinson's Disease: Is it Feasible? Arch Clin Neuropsychol. 2017 Nov 1;32(7):840-860. doi: 10.1093/arclin/acx092.	Different type of study (Review)
Lawrence BJ, Gasson N, Bucks RS, Troeung L, Loftus AM. Cognitive Training and Noninvasive Brain Stimulation for Cognition in Parkinson's Disease: A Meta-analysis. Neurorehabil Neural Repair. 2017 Jul;31(7):597-608. doi: 10.1177/1545968317712468. Epub 2017 Jun 5.	Different type of Study (Meta-Analysis)
Burton CZ, Garnett EO, Capellari E, Chang SE, Tso IF, Hampstead BM, Taylor SF. Combined Cognitive Training and Transcranial Direct Current Stimulation in Neuropsychiatric Disorders: A Systematic Review and Meta-analysis. Biol Psychiatry Cogn Neurosci Neuroimaging. 2023 Feb;8(2):151-161. doi: 10.1016/j.bpsc.2022.09.014. Epub 2022 Oct 13.	Different type of study (Review)
Gillain S, Petermans J. Contribution of new techniques to study the gait in old populations. Ann Phys Rehabil Med. 2013 Jul;56(5):384-95. doi: 10.1016/j.rehab.2013.05.002. Epub 2013 Jun 19.	Different type of study (Review)
Kamble N, Bhattacharya A, Hegde S, Vidya N, Gothwal M, Yadav R, Pal PK. Cortical excitability changes as a marker of cognitive impairment in Parkinson's disease. Behav Brain Res. 2022 Mar 26;422:113733. doi: 10.1016/j.bbr.2022.113733. Epub 2022 Jan 5.	Different type of study (No therapeutical intervention of NIBS)

Schneider JS, Kortagere S. Current concepts in treating mild cognitive impairment in Parkinson's disease. Neuropharmacology. 2022 Feb 1;203:108880. doi: 10.1016/j.neuropharm.2021.108880. Epub 2021 Nov 10.	Different type of study (Review)
Bilgic B, Hanagasi HA, Emre M. Dementia and behavioral neurology: recent advances. J Neurol. 2012 May;259(5):1006-10. doi: 10.1007/s00415-012-6460-3. Epub 2012 Apr 18.	Different type of study (Review)
Di Lazzaro V, Bella R, Benussi A, Bologna M, Borroni B, Capone F, Chen KS, Chen R, Chistyakov AV, Classen J, Kiernan MC, Koch G, Lanza G, Lefaucheur JP, Matsumoto H, Nguyen JP, Orth M, Pascual-Leone A, Rektorova I, Simko P, Taylor JP, Tremblay S, Ugawa Y, Dubbioso R, Ranieri F. Diagnostic contribution and therapeutic perspectives of transcranial magnetic stimulation in dementia. Clin Neurophysiol. 2021 Oct;132(10):2568-2607. doi: 10.1016/j.clinph.2021.05.035. Epub 2021 Jul 20.	Different type of study (Review)
Tang Y, He P, Li Y, Ma G, Zhang Y, Wang L, Nie K. Do Novel Nonexercise and Nondrug Treatments Improve Global Cognition in Parkinson's Disease Patients? A Systematic Review and Bayesian Analysis. Eur J Neurosci. 2025 Feb;61(3):e70008. doi: 10.1111/ejn.70008.	Different type of study (Review)
Firouzi M, Baetens K, Swinnen E, Baeken C, Van Overwalle F, Deroost N. Does transcranial direct current stimulation of the primary motor cortex improve implicit motor sequence learning in Parkinson's disease? J Neurosci Res. 2024 Feb;102(2):e25311. doi: 10.1002/jnr.25311.	Different type of outcomes
Lau CI, Liu MN, Chang KC, Chang A, Bai CH, Tseng CS, Walsh V, Wang HC. Effect of single-session transcranial direct current stimulation on cognition in Parkinson's disease. CNS Neurosci Ther. 2019 Nov;25(11):1237-1243. doi: 10.1111/cns.13210. Epub 2019 Aug 19. PMID: 31424182.	Different type of Population
Pigott JS, Armstrong M, Tabassum N, Davies N, Schrag A. Effectiveness and Feasibility of Nonpharmacological Interventions for People With Parkinson's Disease and Cognitive Impairment on Patient-Centred Outcomes: Systematic Review and Meta-Analysis. Parkinsons Dis. 2024 Nov 18;2024:3654652. doi: 10.1155/2024/3654652.	Different type of study (Review)
Chu CS, Chang HA, Lin YT, Shen HC, Liang CK, Hsu YH, Pan CC, Kuo HY, Liang WZ, Chen SL, Chen CS. Effectiveness and safety of high-definition transcranial direct	Different type of Population

current stimulation in patients with mild cognitive impairment: A randomized, triple-blind, sham-controlled trial. J Alzheimers Dis. 2025 Nov;108(1):298-311. doi: 10.1177/13872877251376547. Epub 2025 Sep 22.	
Zhao Q, Cong G, Lyu H, Zhang Y, Li W, Hu P, Wang K. Effects of low-frequency and high-frequency repetitive transcranial magnetic stimulation combined with levodopa and benserazide hydrochloride on mild cognitive impairment in patients with Parkinson disease. Chin J Behav Med Brain Sci. 2024;33(3):199-205. doi: 10.3760/cma.j.cn371468-20230517-00241.	Not English language
Deng S, Dong Z, Pan L, Liu Y, Ye Z, Qin L, Liu Q, Qin C. Effects of repetitive transcranial magnetic stimulation on gait disorders and cognitive dysfunction in Parkinson's disease: A systematic review with meta-analysis. Brain Behav. 2022 Aug;12(8):e2697. doi: 10.1002/brb3.2697. Epub 2022 Jul 21.	Different type of study (Review)
Saeidi E, Rostami HR, Vahedi M, Haghgoo HA. Effects of transcranial direct current stimulation along with cognitive training on cognitive functions and activities of daily living in patients with Parkinson's disease: A pilot randomized controlled trial. Neurol Clin Neurosci. 2025. doi: 10.1111/ncn3.12841.	Different type of Population
Pagali SR, Kumar R, LeMahieu AM, Basso MR, Boeve BF, Croarkin PE, Geske JR, Hassett LC, Huston J 3rd, Kung S, Lundstrom BN, Petersen RC, St Louis EK, Welker KM, Worrell GA, Pascual-Leone A, Lapid MI. Efficacy and safety of transcranial magnetic stimulation on cognition in mild cognitive impairment, Alzheimer's disease, Alzheimer's disease-related dementias, and other cognitive disorders: a systematic review and meta-analysis. Int Psychogeriatr. 2024 Oct;36(10):880-928. doi: 10.1017/S1041610224000085. Epub 2024 Feb 8.	Different type of Study (Review)
Teselink J, Bawa KK, Koo GK, Sankhe K, Liu CS, Rapoport M, Oh P, Marzolini S, Gallagher D, Swardfager W, Herrmann N, Lanctôt KL. Efficacy of non-invasive brain stimulation on global cognition and neuropsychiatric symptoms in Alzheimer's disease and mild cognitive impairment: A meta-analysis and systematic review. Ageing Res Rev. 2021 Dec;72:101499. doi: 10.1016/j.arr.2021.101499. Epub 2021 Oct 23.	Different type of Study (Review)

Zhang Y, Xu K, Wang Y, Shen Y, Liu Z, Zhang C, Zhou Y, Lv P, Bai Y, Wang S. Efficacy of repetitive transcranial magnetic stimulation in cognitive impairment of neurodegenerative diseases: a systematic review and meta-analysis. BMC Neurol. 2025 Jul 17;25(1):295. doi: 10.1186/s12883-025-04292-4.	Different type of study (Review)
Inoue Y, Sasai T, Hirata K. Electroencephalographic Finding in Idiopathic REM Sleep Behavior Disorder. Neuropsychobiology. 2015;71(1):25-33. doi: 10.1159/000363343. Epub 2015 Feb 27.	Different type of study (Review)
Al-Shamali HF, Janssen-Aguilar R, Hussaini A, Sharma V, Elugot H, et al. Innovative and Emerging Interventional Psychiatry Treatments for the Dual Diagnoses of Substance Use and Psychiatric Disorders: A Systematic Review. Curr Addict Rep. 2025. doi: 10.1007/s40429-025-00685-0.	Different type of study (Review)
Nai YH, Watabe H. Evaluation of the Feasibility of Screening Tau Radiotracers Using an Amyloid Biomathematical Screening Methodology. Comput Math Methods Med. 2018 Dec 19;2018:6287913. doi: 10.1155/2018/6287913.	Different type of Population
Lefaucheur JP, Aleman A, Baeken C, Benninger DH, Brunelin J, Di Lazzaro V, Filipović SR, Grefkes C, Hasan A, Hummel FC, Jääskeläinen SK, Langguth B, Leocani L, Londero A, Nardone R, Nguyen JP, Nyffeler T, Oliveira-Maia AJ, Oliviero A, Padberg F, Palm U, Paulus W, Poulet E, Quartarone A, Rachid F, Rektorová I, Rossi S, Sahlsten H, Schecklmann M, Szekely D, Ziemann U. Evidence-based guidelines on the therapeutic use of repetitive transcranial magnetic stimulation (rTMS): An update (2014-2018). Clin Neurophysiol. 2020 Feb;131(2):474-528. doi: 10.1016/j.clinph.2019.11.002. Epub 2020 Jan 1. Erratum in: Clin Neurophysiol. 2020 May;131(5):1168-1169. doi: 10.1016/j.clinph.2020.02.003.	Different type of study (Guideline)
Liu CS, Herrmann N, Song BX, Ba J, Gallagher D, Oh PI, Marzolini S, Rajji TK, Charles J, Papneja P, Rapoport MJ, Andreazza AC, Vieira D, Kiss A, Lanctôt KL. Exercise priming with transcranial direct current stimulation: a study protocol for a randomized, parallel-design, sham-controlled trial in mild cognitive impairment and Alzheimer's disease. BMC Geriatr. 2021 Dec 4;21(1):677. doi: 10.1186/s12877-021-02636-6.	Different type of Population

Marco-Garcia S, Ferrer-Quintero M, Usall J, Ochoa S, Del Cacho N, Huerta-Ramos E. Facial emotion recognition in neurological disorders: a narrative review. Rev Neurol. 2019 Sep 1;69(5):207-219. Spanish, English. doi: 10.33588/rn.6905.2019047.	Different type of study (Review)
Dubbioso R, Manganelli F, Siebner HR, Di Lazzaro V. Fast Intracortical Sensory-Motor Integration: A Window Into the Pathophysiology of Parkinson's Disease. Front Hum Neurosci. 2019 Apr 8;13:111. doi: 10.3389/fnhum.2019.00111.	Different type of study (Perspective article)
Nardone R, Bergmann J, Brigo F, Christova M, Kunz A, Seidl M, Tezzon F, Trinka E, Golaszewski S. Functional evaluation of central cholinergic circuits in patients with Parkinson's disease and REM sleep behavior disorder: a TMS study. J Neural Transm (Vienna). 2013 Mar;120(3):413-22. doi: 10.1007/s00702-012-0888-6. Epub 2012 Aug 19.	Different type of Population
Manganelli F, Vitale C, Santangelo G, Pisciotta C, Iodice R, Cozzolino A, Dubbioso R, Picillo M, Barone P, Santoro L. Functional involvement of central cholinergic circuits and visual hallucinations in Parkinson's disease. Brain. 2009 Sep;132(Pt 9):2350-5. doi: 10.1093/brain/awp166. Epub 2009 Jul 7.	Different type of Population
Aquino CH, Moscovich M, Marinho MM, Barcelos LB, Felício AC, Halverson M, Hamani C, Ferraz HB, Munhoz RP. Fundamentals of deep brain stimulation for Parkinson's disease in clinical practice: part 1. Arq Neuropsiquiatr. 2024 Apr;82(4):1-9. doi: 10.1055/s-0044-1786026. Epub 2024 Apr 23. PMID: 38653485.	Different type of study (Review)
Brugarolas P, Wilks MQ, Noel J, Kaiser JA, Vesper DR, Ramos-Torres KM, Guehl NJ, Macdonald-Soccorso MT, Sun Y, Rice PA, Yokell DL, Lim R, Normandin MD, El Fakhri G. Human biodistribution and radiation dosimetry of the demyelination tracer [^{18}F]3F4AP. Eur J Nucl Med Mol Imaging. 2023 Jan;50(2):344-351. doi: 10.1007/s00259-022-05980-w. Epub 2022 Oct 5.	Different type of Population
Koo GK, Gaur A, Tumati S, Kusumo RW, Bawa KK, Herrmann N, Gallagher D, Lanctôt KL. Identifying factors influencing cognitive outcomes after anodal transcranial direct current stimulation in older adults with and without cognitive impairment: A systematic review. Neurosci	Different type of Study (Review)

Biobehav Rev. 2023 Mar;146:105047. doi: 10.1016/j.neubiorev.2023.105047. Epub 2023 Jan 14.	
Badr MY, Ahmed GK, Amer RA, Aref HM, Salem RM, Elmokadem HA, Khedr EM. Impact of Repetitive Transcranial Magnetic Stimulation on Cognitive and Psychiatric Dysfunction in Patients with Fibromyalgia: A Double-Blinded, Randomized Clinical Trial. Brain Sci. 2024 Apr 24;14(5):416. doi: 10.3390/brainsci14050416.	Different type of Population
Versace V, Langthaler PB, Sebastianelli L, Höller Y, Brigo F, Orioli A, Saltuari L, Nardone R. Impaired cholinergic transmission in patients with Parkinson's disease and olfactory dysfunction. J Neurol Sci. 2017 Jun 15;377:55-61. doi: 10.1016/j.jns.2017.03.049. Epub 2017 Mar 31.	Different type of Population
Wu X, Hui Y, Wang L, Qiao H, Wang Y, Bai Y, Sun Q, Gao S, Zhang Q, Li L. Long-term intermittent theta burst stimulation alleviates Parkinson's disease-related cognitive impairment by modulating GluN2B in the dorsal hippocampus. Exp Neurol. 2025 Dec;394:115439. doi: 10.1016/j.expneurol.2025.115439. Epub 2025 Aug 21.	Different type of Population
McMackin R, Muthuraman M, Groppa S, Babiloni C, Taylor JP, Kiernan MC, Nasseroleslami B, Hardiman O. Measuring network disruption in neurodegenerative diseases: New approaches using signal analysis. J Neurol Neurosurg Psychiatry. 2019 Sep;90(9):1011-1020. doi: 10.1136/jnnp-2018-319581. Epub 2019 Feb 13	Different type of study (Review)
Manenti R, Brambilla M, Benussi A, Rosini S, Cobelli C, Ferrari C, Petesi M, Orizio I, Padovani A, Borroni B, Cotelli M. Mild cognitive impairment in Parkinson's disease is improved by transcranial direct current stimulation combined with physical therapy. Mov Disord. 2016 May;31(5):715-24. doi: 10.1002/mds.26561. Epub 2016 Feb 16.	Different type of Population
Farina E, Borgnis F, Pozzo T. Mirror neurons and their relationship with neurodegenerative disorders. J Neurosci Res. 2020 Jun;98(6):1070-1094. doi: 10.1002/jnr.24579. Epub 2020 Jan 23.	Different type of study (Review)
Beheshti I, Ko JH. Modulating brain networks associated with cognitive deficits in Parkinson's disease. Mol Med. 2021 Mar 10;27(1):24. doi: 10.1186/s10020-021-00284-5.	Different type of study (Review)

Yang T, Liu W, He J, Gui C, Meng L, Xu L, Jia C. The cognitive effect of non-invasive brain stimulation combined with cognitive training in Alzheimer's disease and mild cognitive impairment: a systematic review and meta-analysis. Alzheimers Res Ther. 2024 Jun 27;16(1):140. doi: 10.1186/s13195-024-01505-9.	Different type of study (Review)
McKiernan E, Su L, O'Brien J. MRS in neurodegenerative dementias, prodromal syndromes and at-risk states: A systematic review of the literature. NMR Biomed. 2023 Jul;36(7):e4896. doi: 10.1002/nbm.4896. Epub 2023 Feb 6.	Different type of study (Review)
da Silva Machado CB, da Silva LM, Gonçalves AF, Andrade PR, Mendes CKTT, de Assis TJCF, Godeiro Júnior CO, Andrade SM. Multisite non-invasive brain stimulation in Parkinson's disease: A scoping review. NeuroRehabilitation. 2021;49(4):515-531. doi: 10.3233/NRE-210190.	Different type of study (Review)
Jellinger KA. Neurobiology of cognitive impairment in Parkinson's disease. Expert Rev Neurother. 2012 Dec;12(12):1451-66. doi: 10.1586/ern.12.131.	Different type of study (Review)
Cole RC, Okine DN, Yeager BE, Narayanan NS. Neuromodulation of cognition in Parkinson's disease. Prog Brain Res. 2022;269(1):435-455. doi: 10.1016/bs.pbr.2022.01.016. Epub 2022 Feb 11.	Different type of study (Review)
Lucero J, Gurnani A, Weinberg J, Shih LC. Neutrophil-to-lymphocyte ratio and longitudinal cognitive performance in Parkinson's disease. Ann Clin Transl Neurol. 2024 Sep;11(9):2301-2313. doi: 10.1002/acn3.52144. Epub 2024 Jul 19.	Different type of Population
Velayudhan L, Ffytche D, Ballard C, Aarsland D. New Therapeutic Strategies for Lewy Body Dementias. Curr Neurol Neurosci Rep. 2017 Sep;17(9):68. doi: 10.1007/s11910-017-0778-2.	Different type of study (Review)
He J, Tang Y, Lin J, Faulkner G, Tsang HWH, Chan SHW. Non-invasive brain stimulation combined with psychosocial intervention for depression: a systematic review and meta-analysis. BMC Psychiatry. 2022 Apr 19;22(1):273. doi: 10.1186/s12888-022-03843-0. PMID: 35439977.	Different type of study (Review)
Biundo R, Fiorenzato E, Antonini A. Nonmotor Symptoms and Natural History of Parkinson's Disease: Evidence From Cognitive Dysfunction and Role of Noninvasive	Different type of study (Review)

Interventions. Int Rev Neurobiol. 2017;133:389-415. doi: 10.1016/bs.irn.2017.05.031. Epub 2017 Jul 13.	
Goldman JG. Non-motor Symptoms and Treatments in Parkinson's Disease. Neurol Clin. 2025 May;43(2):291-317. doi: 10.1016/j.ncl.2024.12.008. Epub 2025 Jan 22.	Different type of Study (Review)
Pupíková M, Rektorová I. Non-pharmacological management of cognitive impairment in Parkinson's disease. J Neural Transm (Vienna). 2020 May;127(5):799-820. doi: 10.1007/s00702-019-02113-w. Epub 2019 Dec 10.	Different type of study (Review)
Garg PK, Lokitz SJ, Truong L, Putegnat B, Reynolds C, Rodriguez L, Nazih R, Nedrelow J, Guardia M, Uffman JK, Garg S, Thornton PS. Pancreatic uptake and radiation dosimetry of 6-[18F]fluoro-L-DOPA from PET imaging studies in infants with congenital hyperinsulinism. PLoS One. 2017 Nov 8;12(11):e0186340. doi: 10.1371/journal.pone.0186340.	Different type of Population
Fasano A, Daniele A, Albanese A. Treatment of motor and non-motor features of Parkinson's disease with deep brain stimulation. Lancet Neurol. 2012 May;11(5):429-42. doi: 10.1016/S1474-4422(12)70049-2.	Different type of study (Review)
Del Felice A, Castiglia L, Formaggio E, Cattelan M, Scarpa B, Manganotti P, Tenconi E, Masiero S. Personalized transcranial alternating current stimulation (tACS) and physical therapy to treat motor and cognitive symptoms in Parkinson's disease: A randomized cross-over trial. Neuroimage Clin. 2019;22:101768. doi: 10.1016/j.nicl.2019.101768. Epub 2019 Mar 18.	Different type of Population
Sohn MN, Brown JC, Sharma P, Ziemann U, McGirr A. Pharmacological adjuncts and transcranial magnetic stimulation-induced synaptic plasticity: a systematic review. J Psychiatry Neurosci. 2024 Feb 15;49(1):E59-E76. doi: 10.1503/jpn.230090.	Different type of study (Review)
Rodríguez-Fernández L, Zorzo C, Arias JL. Photobiomodulation in the aging brain: a systematic review from animal models to humans. Geroscience. 2024 Dec;46(6):6583-6623. doi: 10.1007/s11357-024-01231-y. Epub 2024 Jun 11.	Different type of Population
Wu Y, Kong Q, Li Y, Feng Y, Zhang B, Liu Y, Yu S, Liu J, Cao J, Cui F, Kong J. Potential scalp acupuncture and brain stimulation targets for common neurological disorders:	Different type of Study (Review)

evidence from neuroimaging studies. Chin Med. 2025 May 7;20(1):58. doi: 10.1186/s13020-025-01106-0.	
Li J, You J, Li Z, Zang J, Wu L, Zhao T. Progress and prospects of Parkinson's disease with depression research: A global bibliometric analysis based on CiteSpace. Medicine (Baltimore). 2025 Feb 14;104(7):e41537. doi: 10.1097/MD.0000000000041537.	Different type of study (Bibliometric Analysis)
Feldman HH, Luchsinger JA, Léger GC, Taylor C, Jacobs DM, Salmon DP, Edland SD, Messer K, Revta C, Flowers SA, Jones KS, Koulman A, Yarasheski KE, Verghese PB, Venkatesh V, Zetterberg H, Durant J, Lupo JL, Gibson GE; ADCS BenfoTeam Study Group. Protocol for a seamless phase 2A-phase 2B randomized double-blind placebo-controlled trial to evaluate the safety and efficacy of benfotiamine in patients with early Alzheimer's disease (BenfoTeam). PLoS One. 2024 May 29;19(5):e0302998. doi: 10.1371/journal.pone.0302998. PMID: 38809849.	Different type of Population
Tsutsumi R, Hanajima R, Hamada M, Shirota Y, Matsumoto H, Terao Y, Ohminami S, Yamakawa Y, Shimada H, Tsuji S, Ugawa Y. Reduced interhemispheric inhibition in mild cognitive impairment. Exp Brain Res. 2012 Apr;218(1):21-6. doi: 10.1007/s00221-011-2997-0. Epub 2012 Jan 11.	Different type of Population
Carnahan RM, Chandramouleeshwaran S, Ahsan N, Raymond R, Nobrega JN, Wang W, Fischer CE, Flint AJ, Herrmann N, Kumar S, Lanctôt KL, Mah L, Mulsant BH, Pollock BG, Rajji TK. Relationship of the revised anticholinergic drug scale with cultured cell-based serum anticholinergic activity and cognitive measures in older adults with mild cognitive impairment or remitted depression. Pharmacotherapy. 2025 Jun;45(6):332-340. doi: 10.1002/phar.70022. Epub 2025 May 6. Erratum in: Pharmacotherapy. 2025 Dec;45(12):863. doi: 10.1002/phar.70077.	Different type of Population
Martini DN, Morris R, Madhyastha TM, Grabowski TJ, Oakley J, Hu SC, Zabetian CP, Edwards KL, Hiller A, Chung K, Ramsey K, Lapidus JA, Cholerton B, Montine TJ, Quinn JF, Horak FB. Relationships Between Sensorimotor Inhibition and Mobility in Older Adults With and Without Parkinson's Disease. J Gerontol A Biol Sci Med Sci. 2021 Mar 31;76(4):630-637. doi: 10.1093/gerona/glaa300.	Different type of Population

He PK, Wang LM, Chen JN, Zhang YH, Gao YY, Xu QH, Qiu YH, Cai HM, Li Y, Huang ZH, Feng SJ, Zhao JH, Ma GX, Nie K, Wang LJ. Repetitive transcranial magnetic stimulation (rTMS) fails to improve cognition in patients with parkinson's disease: a Meta-analysis of randomized controlled trials. Int J Neurosci. 2022 Mar;132(3):269-282. doi: 10.1080/00207454.2020.1809394. Epub 2020 Nov 18.	Different type of Study (Meta-Analysis)
Espiritu AI, Hara T, Tolledo JK, Blair M, Burhan AM. Repetitive transcranial magnetic stimulation for apathy in patients with neurodegenerative conditions, cognitive impairment, stroke, and traumatic brain injury: a systematic review. Front Psychiatry. 2023 Nov 15;14:1259481. doi: 10.3389/fpsyt.2023.1259481.	Different type of Study (Review)
Randver R. Repetitive transcranial magnetic stimulation of the dorsolateral prefrontal cortex to alleviate depression and cognitive impairment associated with Parkinson's disease: A review and clinical implications. J Neurol Sci. 2018 Oct 15;393:88-99. doi: 10.1016/j.jns.2018.08.014. Epub 2018 Aug 15.	Different type of Study (Review)
Rektorova I. Resting-state networks in Alzheimer's disease and Parkinson's disease. Neurodegener Dis. 2014;13(2-3):186-8. doi: 10.1159/000354237. Epub 2013 Sep 4.	Different type of study (Review)
Kim T, Kang DW, Salazar Fajardo JC, Jang H, Um YH, Kim S, Wang SM, Kim D, Lim HK. Safety and feasibility of optimized transcranial direct current stimulation in patients with mild cognitive impairment due to Alzheimer's disease: a multicenter study protocol for a randomized controlled trial. Front Neurol. 2024 Apr 10;15:1356073. doi: 10.3389/fneur.2024.1356073.	Different type of Population
Cao J, Chai-Zhang TC, McDonald CM, Kong J. Scalp Stimulation Targets for Neurological Conditions-Evidence from Large-Scale Meta-Analyses. J Integr Neurosci. 2022 Apr 15;21(3):83. doi: 10.31083/j.jin2103083.	Different type of study (Meta-Analysis)
Yarnall AJ, Rochester L, Baker MR, David R, Khoo TK, Duncan GW, Galna B, Burn DJ. Short latency afferent inhibition: a biomarker for mild cognitive impairment in Parkinson's disease? Mov Disord. 2013 Aug;28(9):1285-8. doi: 10.1002/mds.25360. Epub 2013 Feb 28.	Different type of study (No NIBS therapeutical intervention)
Yang D, Shin YI, Hong KS. Systemic Review on Transcranial Electrical Stimulation Parameters and EEG/fNIRS Features	Different type of study (Review)

for Brain Diseases. Front Neurosci. 2021 Mar 26;15:629323. doi: 10.3389/fnins.2021.629323.	
Cankaya S, Akturk A, Karakus A, Hanoğlu L, Mardinoglu A, Yulug B. Targeting the parietal memory network with tDCS in MCI: study protocol for a randomized controlled trial. Front Hum Neurosci. 2025 Nov 10;19:1661790. doi: 10.3389/fnhum.2025.1661790.	Different type of Population
Flöel A. tDCS-enhanced motor and cognitive function in neurological diseases. Neuroimage. 2014 Jan 15;85 Pt 3:934-47. doi: 10.1016/j.neuroimage.2013.05.098. Epub 2013 May 30.	Different type of study (Review)
Huang H, Bach JR, Sharma HS, Saberi H, Jeon SR, et al. The 2022 yearbook of neurorestoratology. J Neurorestoratol. 2023. doi: 10.1016/j.jnrt.2023.100054.	Different type of study (Review)
Cantone M, Di Pino G, Capone F, Piombo M, Chiarello D, Cheeran B, Pennisi G, Di Lazzaro V. The contribution of transcranial magnetic stimulation in the diagnosis and in the management of dementia. Clin Neurophysiol. 2014 Aug;125(8):1509-32. doi: 10.1016/j.clinph.2014.04.010. Epub 2014 Apr 30.	Different type of study (Review)
Zheng B, Chen J, Cao M, Zhang Y, Chen S, Yu H, Liang K. The effect of intermittent theta burst stimulation for cognitive dysfunction: a meta-analysis. Brain Inj. 2024 Jul 28;38(9):675-686. doi: 10.1080/02699052.2024.2344087. Epub 2024 Apr 23.	Different type of Study (Meta-Analysis)
Han BH, Wallner K, Merrick G, Badiozamani K, Butler W. The effect of interobserver differences in post-implant prostate CT image interpretation on dosimetric parameters. Med Phys. 2003 Jun;30(6):1096-102. doi: 10.1118/1.1576232.	Different type of Population
Khedr EM, Mohamed KO, Ali AM, Hasan AM. The effect of repetitive transcranial magnetic stimulation on cognitive impairment in Parkinson's disease with dementia: Pilot study. Restor Neurol Neurosci. 2020;38(1):55-66. doi: 10.3233/RNN-190956.	Different type of Population
Ferrazzano G, Maccarrone D, Guerra A, Collura A, Satriano F, Fratino M, Ievolella F, Belvisi D, Amato MP, Centonze D, Altieri M, Conte A, Leodori G. The effects of gamma-tACS on cognitive impairment in multiple sclerosis: A randomized, double-blind, sham-controlled, pilot study.	Different type of Population

Mult Scler. 2025 May;31(6):728-739. doi: 10.1177/13524585251333575. Epub 2025 Apr 26.	
Wu YJ, Tseng P, Huang HW, Hu JF, Juan CH, Hsu KS, Lin CC. The Facilitative Effect of Transcranial Direct Current Stimulation on Visuospatial Working Memory in Patients with Diabetic Polyneuropathy: A Pre-post Sham-Controlled Study. Front Hum Neurosci. 2016 Sep 28;10:479. doi: 10.3389/fnhum.2016.00479.	Different type of Population
Rektor I, Bočková M, Chrastina J, Rektorová I, Baláž M. The modulatory role of subthalamic nucleus in cognitive functions - a viewpoint. Clin Neurophysiol. 2015 Apr;126(4):653-8. doi: 10.1016/j.clinph.2014.10.156. Epub 2014 Nov 8.	Different type of study (Opinion)
Turner TH, Scott EP, Barlis K, Rodriguez-Porcel F, Sartori AC, Joseph J. The Rapid Access Memory Program for Addressing Concerns of Incipient Dementia in Academic Primary Care Settings. J Geriatr Psychiatry Neurol. 2024 Jul;37(4):255-262. doi: 10.1177/08919887231225482. Epub 2023 Dec 29.	Different type of Population
Hanoğlu T, Hanoğlu L, Güntekin B, Aktürk T, Yulug B. The therapeutic role of repetitive transcranial magnetic stimulation (rTMS) in parkinsonian visual hallucinations: Electrophysiological correlates. J Clin Neurosci. 2019 Nov;69:281-284. doi: 10.1016/j.jocn.2019.08.002. Epub 2019 Aug 30.	Different type of Population
Argon M, Secil Y, Duygun U, Aydogdu I, Kocacelebi K, Ozkilic H, Ertekin C. The value of scintigraphy in the evaluation of oropharyngeal dysphagia. Eur J Nucl Med Mol Imaging. 2004 Jan;31(1):94-8. doi: 10.1007/s00259-003-1276-0. Epub 2003 Oct 22.	Different type of Population
Giustiniani A, Maistrello L, Mologni V, Danesin L, Burgio F. TMS and tDCS as potential tools for the treatment of cognitive deficits in Parkinson's disease: a meta-analysis. Neurol Sci. 2025 Feb;46(2):579-592. doi: 10.1007/s10072-024-07778-0. Epub 2024 Sep 25.	Different type of study (Meta-Analysis)
Mantovani E, Bressan MM, Tinazzi M, Tamburin S. Towards multimodal cognition-based treatment for cognitive impairment in Parkinson's disease: drugs, exercise, non-invasive brain stimulation and technologies. Curr Opin	Different type of study (Review)

Neurol. 2024 Dec 1;37(6):629-637. doi: 10.1097/WCO.0000000000001310. Epub 2024 Jul 30.	
Brak IV, Filimonova E, Zakhariya O, Khasanov R, Stepanyan I. Transcranial Current Stimulation as a Tool of Neuromodulation of Cognitive Functions in Parkinson's Disease. Front Neurosci. 2022 Jul 12;16:781488. doi: 10.3389/fnins.2022.781488.	Different Type of Study (Review)
Adenzato M, Manenti R, Enrici I, Gobbi E, Brambilla M, Alberici A, Cotelli MS, Padovani A, Borroni B, Cotelli M. Transcranial direct current stimulation enhances theory of mind in Parkinson's disease patients with mild cognitive impairment: a randomized, double-blind, sham-controlled study. Transl Neurodegener. 2019 Jan 7;8:1. doi: 10.1186/s40035-018-0141-9.	Different type of outcomes
Firouzi M, Van Herk K, Kerckhofs E, Swinnen E, Baeken C, Van Overwalle F, Deroost N. Transcranial direct-current stimulation enhances implicit motor sequence learning in persons with Parkinson's disease with mild cognitive impairment. J Neuropsychol. 2021 Sep;15(3):363-378. doi: 10.1111/jnp.12231. Epub 2020 Nov 18.	Different type of outcomes
Padovani A, Benussi A, Cotelli MS, Ferrari C, Cantoni V, Dell'Era V, Turrone R, Paghera B, Borroni B. Transcranial magnetic stimulation and amyloid markers in mild cognitive impairment: impact on diagnostic confidence and diagnostic accuracy. Alzheimers Res Ther. 2019 Dec 1;11(1):95. doi: 10.1186/s13195-019-0555-3.	Different type of Population
Elder GJ, Taylor JP. Transcranial magnetic stimulation and transcranial direct current stimulation: treatments for cognitive and neuropsychiatric symptoms in the neurodegenerative dementias? Alzheimers Res Ther. 2014 Nov 10;6(9):74. doi: 10.1186/s13195-014-0074-1.	Different type of Study (Review)
Antczak J, Rusin G, Słowik A. Transcranial Magnetic Stimulation as a Diagnostic and Therapeutic Tool in Various Types of Dementia. J Clin Med. 2021 Jun 28;10(13):2875. doi: 10.3390/jcm10132875.	Different type of study (Review)
Dognini E, Finazzi S, Campana E, Manenti R, Cotelli M, Borroni B. Transcranial Magnetic Stimulation as a Diagnostic Tool in Mild Cognitive Impairment: A Systematic Review. Brain Sci. 2025 Sep 9;15(9):969. doi: 10.3390/brainsci15090969.	Different type of study (Review)

Somaa FA, de Graaf TA, Sack AT. Transcranial Magnetic Stimulation in the Treatment of Neurological Diseases. Front Neurol. 2022 May 20;13:793253. doi: 10.3389/fneur.2022.793253.	Different type of study (Review)
García-Castro P, Conejo NM, González-Pardo H. Transcranial photobiomodulation therapy in older women regarding cognitive functions: a systematic review. Lasers Med Sci. 2025 Nov 15;40(1):480. doi: 10.1007/s10103-025-04742-y.	Different type of study (Review)
Sun C, Armstrong MJ. Treatment of Parkinson's Disease with Cognitive Impairment: Current Approaches and Future Directions. Behav Sci (Basel). 2021 Apr 17;11(4):54. doi: 10.3390/bs11040054.	Different type of study (Review)
Liu CS, Rau A, Gallagher D, Rajji TK, Lanctôt KL, Herrmann N. Using transcranial direct current stimulation to treat symptoms in mild cognitive impairment and Alzheimer's disease. Neurodegener Dis Manag. 2017 Oct;7(5):317-329. doi: 10.2217/nmt-2017-0021. Epub 2017 Oct 18.	Different type of Study (Review)
