# Supplementary material for: The Use of Non-Invasive Brain Stimulation Techniques in Subjects with Parkinson’s Disease and Mild Cognitive Impairment: A Systematic Review
Source: Brain Sci. 2026 Mar 19;16(3):325. doi: 10.3390/brainsci16030325 (PMC13024718; doi:10.3390/brainsci16030325)
Supplement: Supplementary file 1 [file brainsci-16-00325-s001.zip › File S4. systematic review databases search queries.pdf]

- **PubMed**: (Parkinson OR PD OR “Parkinson’s Disease”) AND (NIBS OR “Non Invasive Brain Stimulation” OR TMS OR “Transcranial Magnetic Stimulation” OR tES OR “Transcranial Electrical Stimulation” OR tDCS OR “Transcranial Direct Current Stimulation” OR tACS OR “Transcranial Alternating Current Stimulation” OR tRNS OR “Transcranial Random Noise Stimulation”) AND (MCI OR “Mild Cognitive Impairment”).
- **Scopus**: TITLE-ABS-KEY (("Parkinson’s disease" OR Parkinson) AND ("non invasive brain stimulation" OR NIBS OR TMS OR "transcranial magnetic stimulation" OR tDCS OR "transcranial direct current stimulation" OR tACS OR "transcranial alternating current stimulation" OR tRNS OR "transcranial random noise stimulation") AND ("mild cognitive impairment" OR MCI)) AND (LIMIT-TO (DOCTYPE , "ar") OR LIMIT-TO (DOCTYPE , "re")) AND (LIMIT-TO (SUBJAREA , "MEDI") OR LIMIT-TO (SUBJAREA , "NEUR") OR LIMIT-TO (SUBJAREA , "PSYC")).
- **Web of Science**: (Parkinson OR PD OR “Parkinson’s Disease”) AND (NIBS OR “Non Invasive Brain Stimulation” OR TMS OR “Transcranial Magnetic Stimulation” OR tES OR “Transcranial Electrical Stimulation” OR tDCS OR “Transcranial Direct Current Stimulation” OR tACS OR “Transcranial Alternating Current Stimulation” OR tRNS OR “Transcranial Random Noise Stimulation”) AND (MCI OR “Mild Cognitive Impairment”).
- **Medline Ultimate**: (Parkinson OR PD OR “Parkinson’s Disease”) AND (NIBS OR “Non Invasive Brain Stimulation” OR TMS OR “Transcranial Magnetic Stimulation” OR tES OR “Transcranial Electrical Stimulation” OR tDCS OR “Transcranial Direct Current Stimulation” OR tACS OR “Transcranial Alternating Current Stimulation” OR tRNS OR “Transcranial Random Noise Stimulation”) AND (MCI OR “Mild Cognitive Impairment”).
